# Functional verification and characterization of a type-III geranylgeranyl diphosphate synthase gene from *Sporobolomyces pararoseus* NGR

**DOI:** 10.3389/fmicb.2022.1032234

**Published:** 2022-11-24

**Authors:** Jianyu Yan, Chunji Li, Ning Zhang, Chunwang Li, Yunjiao Wang, Bingxue Li

**Affiliations:** ^1^College of Bioscience and Biotechnology, Shenyang Agricultural University, Shenyang, China; ^2^Innovative Institute for Plant Health, Zhongkai University of Agriculture and Engineering, Guangzhou, China; ^3^College of Agriculture and Biology, Zhongkai University of Agriculture and Engineering, Guangzhou, China; ^4^College of Land and Environment, Shenyang Agricultural University, Shenyang, China

**Keywords:** *Sporobolomyces pararoseus*, geranylgeranyl diphosphate synthase (GGPPS), *in vitro* enzymatic activity, functional verification, carotenoids

## Abstract

Carotenoids, a group of natural pigments, have strong antioxidant properties and act as precursors to vitamin A, which have garnered attention from industry and researchers. *Sporobolomyces pararoseus* represents a hyper-producer of carotenoids, mainly including β–carotene, torulene, and torularhodin. Geranylgeranyl diphosphate synthase (GGPPS) is regarded as a key enzyme in the carotenoid biosynthesis pathway. However, the precise nature of the gene encoding GGPPS in *S. pararoseus* has not been reported yet. Here, we cloned a cDNA copy of the GGPPS protein-encoding gene *crtE* from *S. pararoseus* NGR. The *crtE* full-length genomic DNA and cDNA are 1,722 and 1,134 bp, respectively, which consist of 9 exons and 8 introns. This gene encodes 377 amino acids protein with a predicted molecular mass of 42.59 kDa and a PI of 5.66. Identification of the *crtE* gene encoding a functional GGPPS was performed using heterologous complementation detection in *Escherichia coli*. *In vitro* enzymatic activity experiments showed that CrtE utilized farnesyl diphosphate (FPP) as an allylic substrate for the condensation reaction with isopentenyl diphosphate (IPP), generating more of the unique product GGPP compared to other allylic substrates. The predicted CrtE 3D-model was analyzed in comparison with yeast GGPPS. The condensation reaction occurs in the cavity of the subunit, and three bulky amino acids (Tyr110, Phe111, and His141) below the cavity prevent further extension of the product. Our findings provide a new source of genes for carotenoid genetic engineering.

## Introduction

Carotenoids are lipophilic isoprenoids found in plenty of bacteria, fungi, algae, and plants. They can be divided into C_30_, C_40_, and C_50_ carotenoids according to the amount of carbon (Yang et al., 2015). In recent years, most of carotenoids have been found to prevent cancer and reduce the risk of heart disease and cataracts and thus widely used in food, cosmetics, health care products, feed, and other industries (Ames et al., 1993; Mussagy et al., 2019). For instance, lycopene prevents lung cancer and cardiovascular diseases (Costa-Rodrigues et al., 2018; Mustra Rakic and Wang, 2020), and astaxanthin protect the brain from oxidative damage as well as prevent obesity (Cakir et al., 2020; Ojulari et al., 2020). Torulene and torularhodin, two monocyclic carotenoids, exhibits more strong antioxidant activities than that of β-carotene, thanks to the existence of thirteen double bonds and a longer polyene chain (Kot et al., 2016). However, due to the extremely unstable structure and few sources of synthesis, more potential functions of torulene and torularhodin have not been explored (Kot et al., 2018).

*S. pararoseus* is recognized as a kind of oleaginous red yeast, belongs to the order Sporidiobolales. This species produces a variety of carotenoids, mainly including β-carotene, torulene, and torularhodin (Li et al., 2016; Li et al., 2020). It can be used as a source of carotenoids for industrial production because of its low cost, short life cycle and lack of environmental restrictions compared to plants (Chaiyaso and Manowattana, 2018). In recent years, there has been increasing research on carotenoids using the strains of *S. pararoseus*, especially in torulene and torularhodin. The salt stress resulted in a significant increase in torulene and torularhodin production (Li et al., 2017). The proportion of torulene in total carotenoids increased when glucose was replaced by citrus juice as the carbon source (Wei et al., 2020). However, as the biosynthetic pathway of *S. pararoseus* carotenoids is progressively discovered, it will be possible to increase carotenoid production through genetic engineering in the future.

Previous studies reported that carotenoids are usually synthesized through the mevalonate (MEV) pathway in oleaginous red yeasts (Hara et al., 2014). Of which, IPP isomerase catalyzed the conversion from isopentenyl diphosphate (IPP) to dimethylallyl diphosphate (DMAPP); geranylgeranyl diphosphate synthase (GGPPS) catalyzed the condensation of DMAPP with three molecules of IPP to form GGPP (Shen et al., 2016; Nagel et al., 2019). Two GGPP units are condensed by CrtYB to form phytoene, which is then dehydrogenated by the desaturase CrtI in four- or five-steps to form lycopene or 3,4-dedihydrolycopene, respectively (Li et al., 2016). Terminals of lycopene are consecutively cyclized by CrtYB to form γ-carotene and β-carotene (Moise et al., 2014). 3,4-dihydrolycopene also could be transformed into torulene and torularhodin through a series of cyclization, hydroxylation, and oxidation reaction (Li et al., 2017). Particularly, the GGPP is a unique precursor for the synthesis of phytoene which is the first pigment in the carotenoids synthesis pathway, hence the flux of GGPP determines the productivity of total carotenoids (Breitenbach et al., 2011). Therefore, elucidating the function of GGPPS in *S. pararoseus* is essential for understanding the entire carotenoid biosynthesis pathway.

GGPPS is a member of the short chain prenyltransferase family. This family also includes other enzymes with the same product chain length extension mechanism such as geranyl diphosphate synthase (GPPS), farnesyl diphosphate synthase (FPPS), and GGPPS that produce C_10_, C_15_, and C_20_ isoprenoids, respectively. The GGPPS were classified into three types based on amino acid sequence characteristics: type I GGPPS in archaea; type II GGPPS in plants and bacteria; type III GGPPS in animals and fungi (Vandermoten et al., 2009). In general, type I and type II GGPPS mainly catalyze the condensation of DMAPP or GPP with IPP to produce GGPP, while the type III GGPPS mainly condense one molecule FPP and IPP to synthesize GGPP (Barbar et al., 2013). Moreover, the isoprenoid diphosphate synthase from *Picea abies*, was found to catalyze the condensation of DMAPP and IPP to produce both GPP and GGPP products (Schmidt et al., 2010). The type of GGPPS needs to be judged based on sequence homology and *in vivo* or *in vitro* assays.

In the present study, we cloned GGPPS-encoding gene *crtE* from *S. pararoseus* NGR and performed functional verification by the combination of *in vivo* and *in vitro* experiments. These findings would enhance our understanding of carotenoids synthetic pathway in *S. pararoseus* NGR and unveil new gene target to further improve its carotenoids production via genetic engineering.

## Materials and methods

### Strains and culture conditions

*S. pararoseus* NGR was isolated from strawberry fruit in the green house of Shenyang Agricultural University (41°49′N, 123°34′E). The strain number is recorded in the China General Microbiological Culture Collection Center as CGMCC 2.5280. *S. pararoseus* NGR was incubated in YPD medium (10 g/L yeast extract, 20 g/L peptone, 20 g/L dextrose, pH 6.0) in shaking flasks at 28°C. *Escherichia coli* DH5α and BL21 (DE3) strains for the cloning and expression of recombinant plasmids were purchased from TIANGEN Bio. Co., Ltd. (Beijing, China). *E. coli* DH5α and BL21 (DE3) strains were incubated at shaking flasks at 37°C in LB medium (10 g/L peptone, 5 g/L yeast extract, 10 g/L NaCl, pH 7.0).

### Gene cloning of putative *crtE* from *S. pararoseus* NGR

Fresh cells of *S. pararoseus* NGR were collected by centrifugation after 3 days of culture in liquid YPD medium (17,000 × g, 1 min, 4°C), and genomic DNA was prepared by the Yeast Genomic DNA Extraction Kit (Solarbio, Beijing, China). Then, total RNA was extracted using the Spin Column Fungal Total RNA Purification Kit (Sangon, Shanghai, China). The total cDNA of *S. pararoseus* NGR was prepared by the PrimeScript II Ist Stand cDNA Synthesis Kit (Takara, Dalian, China), and was stored at −20°C for subsequent experiments.

According to the complete GGPPS gene sequence of *S. pararoseus* NGR resulting from transcriptome sequencing, a pair of specific primers (forward primer *crtE*-F and reverse *crtE*-R, listed in Table 1) were designed to clone the *crtE* with restriction enzymes sites for *Kpn*I and *Bam*HI. The 2 × High Fidelity PCR Master Mix (Sangon, Shanghai, China) was used to amplify *crtE* from genomic DNA or cDNA of *S. pararoseus* NGR, and the reaction procedure was as following: 1 cycle of 95°C for 3 min; 35 cycles of 95°C for 15 s, 55°C for 15 s, and 72°C for 1 min; at last, 72°C for 5 min. The amplified fragment was ligated into pMD18-T vector (Takara, Dalian, China) and transformed into *E. coli* DH5α, then extracted the plasmid pMD18T-*crtE* by TIANprep Mini Plasmid Kit (Tiangen Biotech Co., Ltd., Beijing, China) and sequenced before the further experiments.

**TABLE 1:** Primer sequences used in this study.

Primers	Sequences	Restriction enzymes
*crtE*-F	GGGGTACCATGACGGAGTTCTACGACAACTTTC	*Kpn*I
*crtE*-R	CGGGATCCTCAGTGAGTATCGTTTGTACCATTCG	*Bam*HI
*NGR26S*-F	CGAGCTCTAAGCGGAGGAAAAGAAACTAAC	*Sac*I
*NGR26S*-R	CGAGCTCCGTGGATAAGCCGAAGC	*Sac*I
*crtE*-ori-F	GGGGTACCATGGTGAGTGGCAGTAAAGCG	*Kpn*I
*crtE*-ori-R	CCCAAGCTTTCAGGCGATTTTCATGACCG	*Hin*dIII

Underlined is the sequence of the introduced digestion site. All primers were synthesized by Sangon, Shanghai, China.

### Bioinformatics analysis

Sequence alignment of *crtE* cDNA with its genomic DNA was performed using DNAMAN. The basic physicochemical properties of CrtE were predicted in the ProtParam online tool^1^ with default parameters. Protein sequence alignment was performed according to the ClustalW algorithm with default parameters. Protein secondary structure prediction using the SPOPMA online tool. The three-dimensional structure of CrtE was predicted on the SWISS-MODEL online tool,^2^ and the resulting CrtE model was visualized using the software Pymol (The PyMOL Molecular Graphics System, Version 2.0 Schrödinger, LLC.).

### Construction of plasmids for *crtE* gene expression in *E. coli*

Plasmids pMD18T-*crtE* and pET32a were digested with restriction enzymes *Kpn*I and *Bam*HI (Takara, Dalian, China). The target gene fragment and pET32a linear fragment were ligated with T4 DNA ligase (Takara, Dalian, China) to construct the recombinant plasmid pET32a-*crtE*.

pET32a-*crtE* was transformed into BL21 (DE3) competent cells and pET32a empty vector was used as control. The BL21 (DE3) strain containing plasmids pET32a-*crtE* and pET32a, respectively, were cultured for 12 h, seeded in LB medium (containing 50 μg/mL ampicillin) at 1:20 and incubated at 37°C with shaking flasks until OD_600_ = 0.6, then added with isopropyl β-D-thiogalactoside (IPTG) at final concentration of 1 mM for 4–5 h induction. The cells were centrifuged at 17,000 × g for 1 min, and the supernatant was discarded. The precipitates were washed twice with phosphate buffered saline (PBS) buffer. The precipitates were suspended with non-denatured cracking solution (50 mM NaH_2_PO_4_, 300 mM NaCl, 10 mM imidazole, pH8.0) and added PMSF (Yuanye Bio-Technology, Shanghai, China) with a final concentration of 1 mM. The suspending cells were broken on the ultrasonic cell crusher to release internal solutes then centrifuged at 21,000 × g for 30 min. After centrifugation, the supernatant and precipitation were used as samples for Sodium Dodecyl Sulfate-PolyAcrylamide Gel Electrophoresis (SDS-PAGE) to analyze the expression of CrtE and total protein.

### Enzymatic activity detection

To obtain purified CrtE proteins, the supernatant after cell crushing was subjected to HisTrap HP column (Cytiva, Marlborough, USA), and washed with five column volumes of wash solution (50 mM NaH_2_PO_4_, 300 mM NaCl, 20 mM imidazole, pH 8.0), and eluted with five column volumes of elution solution (50 mM NaH_2_PO_4_, 300 mM NaCl, 500 mM imidazole, pH 8.0). The resulting purified CrtE was concentrated by Amicon Ultra-0.5 Centrifugal Filter (10 kDa) (Millipore, Billerica, MA, USA) and exchanged with 100 mM HEPES buffer (containing 5 mM MgCl_2_, pH7.5). Protein concentrations were determined by the Bradford Protein Assay Kit (Solarbio, Beijing, China).

Addition of 10 ng CrtE protein, 50 μM allylic substrate (DMAPP, GPP, or FPP) and 50 μM IPP to the enzymatic activity reaction system and made up to 200 μL with 100 mM HEPES buffer. DMAPP, GPP, FPP, and IPP were purchased from Sigma-Aldrich (Sigma-Aldrich, St Louis, USA). The reaction was incubated at 28°C for 2 h. Then added 200 μL Tris-HCl (pH 9.5) buffer containing 2 U SAP (Shrimp Alkaline Phosphatase) (Takara, Dalian, China) and 2 U TIPP (Thermostable Inorganic Pyrophosphatase) (NEB, USA), and reacted for 12 h at 30°C for dephosphorylation of the product. The reactions were stopped on ice, and added 400 μL hexane for extraction, repeated twice. Then the hexane layer was collected and evaporated to 100 μL under a N_2_ stream, stored at −80°C for thin layer chromatography (TLC) analysis.

The above samples collected in hexane were analyzed by TLC in TLC Silica gel 60 F_254_ (Merck, Germany), and GOH, FOH, and GGOH mixtures were used as standards, developed with toluene/acetonitrile/ethylacetate/acetic acid (35:5:15:0.15, v/v/v/v). The standards GOH, FOH, and GGOH were purchased from Sigma-Aldrich (Sigma-Aldrich, St Louis, USA). Color development was performed with iodine vapor and the product distribution was observed by TLC scanner KH-3100 (KEZHE, Shanghai, China).

### Functional analysis of putative *crtE* gene

**FIGURE 1:**
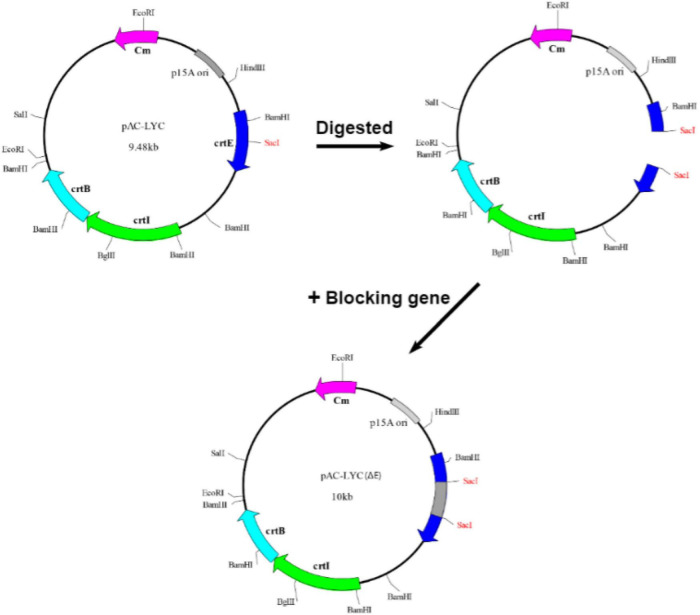
Construction of pAC-LYC (Δ*E*). A sequence not coding a functional protein was inserted into the pAC-LYC plasmid at the *Sac*I position of the *crtE* gene. The *crtE* gene was rendered incapable of expressing functional proteins, but did not affect the expression of the *crtB* or *crtI* genes.

The following protocol was used for *in vivo* functional analysis of the putative *crtE* gene. Plasmid pAC-LYC carries three carotenogenic genes *crtE*, *crtB*, and *crtI* from *Erwinia herbicola* and can synthesize lycopene in *E. coli*. As shown in Figure 1, a non-functional *S. pararoseus* strain NGR large subunit ribosomal RNA sequence (*26S*) (GenBank accession number ON197886) was inserted into the *Sac*I site of pAC-LYC to construct plasmid pAC-LYC (Δ*E*). Since *E. coli* cannot synthesize GGPP by itself (Umeno et al., 2005), the plasmid pAC-LYC (Δ*E*) cannot synthesize lycopene in *E. coli* and therefore behaves as white. The plasmids mentioned above are shown in Table 2. Plasmid pET32a-*crtE* was transformed into the *E. coli* BL21 (DE3) carrying the plasmid pAC-LYC (Δ*E*), pET32a empty vector co-expressed with pAC-LYC (Δ*E*) in BL21 (DE3) as a control strain. Recombinant strains were incubated in LB medium (containing 50 μg/mL ampicillin and 25 μg/mL chloramphenicol) at 37°C until OD_600_ = 0.6. IPTG with a final concentration of 1 mM was added for induction and cultured in dark for 48 h at 37°C. The cells were collected by centrifugation (11,500 × g, 10 min) to observe the color of recombinant *E. coli* as a preliminary identification. Lastly, qualitative and quantitative analysis of carotenoids were conducted using high performance liquid chromatography (HPLC) method.

**TABLE 2:** Plasmids used in this study and their main characteristics.

Plasmids	Genes	Resistent
pET32a (+)	None	Ampicillin
pAC-LYC	*crtE, crtI, crtB*	Chloramphenicol
pMD18T-*crtE*	*crtE*	Ampicillin
pET32a-*crtE*	*crtE*	Ampicillin
pAC-LYC (Δ*E*)	*crtI, crtB*	Chloramphenicol

### Carotenoid extraction and detection

Added 10 mL of dimethyl sulfoxide to the *E. coli* cells collected from 1 L of culture medium, mixed thoroughly, and treated in a water bath at 65°C for 30 min, shaking several times during the period. Then added 20 mL acetone and stranded 20 min, the pigment supernatant was collected by centrifugation (16,000 × g, 1 min). Sucked the supernatant and mixed it with 15 mL hexane, let it stood for a moment, and took the hexane layer containing pigment. The hexane layer was concentrated under nitrogen before HPLC detection. Carotenoids were detected using the Agilent 1290 Infinity II LC system (Agilent Technologies, Palo Alto, CA, USA) equipped with a reverse phase C_18_ column (5 μm, 150 × 4.6 mm) (Thermo Fisher Scientific, Waltham, MA, USA) with an isocratic solvent system consisting of acetonitrile/methanol/isopropanol (85:10:5, v/v/v) at a flow rate of 1 mL/min, an injection volume of 20 μL, a column temperature of 32°C and a wavelength of 450 nm. Lycopene standard was purchased from Meilun Biotech Co., Ltd. (Dalian, China).

## Results

### Putative geranylgeranyl diphosphate synthase gene (*crtE*) cloning and sequence analysis

**FIGURE 2:**
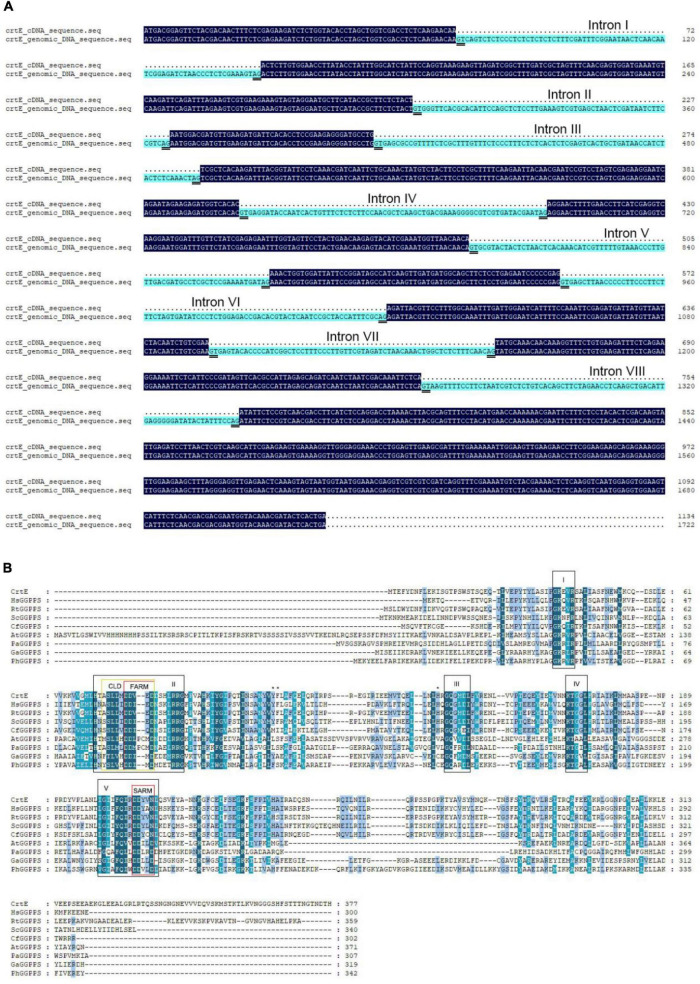
Sequences alignment. **(A)** Alignment of the *crtE* cDNA sequence with genomic DNA sequence of *S. pararoseus* NGR. **(B)** Alignment were made between geranylgeranyl diphosphate synthetase amino acid sequences in different species. Abbreviations and accession numbers: CrtE, *Sporobolomyces pararoseus* GGPPS, AVN68390.1; HsGGPPS, Human GGPPS, BAA75909.1; RtGGPPS, *Rhodotorula toruloides* GGPPS, BBE10611.1; ScGGPPS, *Saccharomyces cerevisiae* GGPPS, 2E8T_A; CfGGPPS, *Choristoneura fumiferana* GGPPS, AGW99945.1; AtGGPPS, *Arabidopsis thaliana* GGPPS, AAA32797.1; PaGGPPS, *Pantoea agglomerans* pyrophosphate synthase, AAA64977.1; GaGGPPS, *Geoglobus acetivorans* GGPPS, WP_048092150.1; PhGGPPS, *Pyrococcus horikoshii* GGPPS, WP_010885159.1. Conserved sequence motifs found in GGPPS are labeled as I–V and highlighted with black boxes. Domains II and V contain first aspartic acid-rich motif (FARM) and second aspartic acid-rich motif (SARM), respectively, both highlighted by red boxes. The region which contains the FARM and the five amino acids upstream is the chain length determination region (CLD), highlighted by a yellow box. The three bulky amino acids Tyr110, Phe111, and His141) mentioned in the main text are marked with an asterisk (*).

In order to clone the putative geranylgeranyl diphosphate synthase gene (*crtE*) from *S. pararoseus* NGR, specific primers, *crtE*-F and *crtE*-R, were designed based on the transcriptome data. We obtained a full-length cDNA and genomic DNA clone of the *crtE* gene, 1,134 and 1,722 bp, respectively. Comparison of the two sequences showed that the *crtE* genomic DNA contains eight introns and all splicing sites following the GT-AG rule (Figure 2A). The corresponding full length cDNA clone contains a complete open reading frame (ORF), encodes a polypeptide with 377 amino acids that was predicted in the ProtParam Tool to have a protein molecular weight is 42.59 kDa and the isoelectric point is 5.66. The cDNA sequences and deduced amino acids of *crtE* reported in this study are available in the GenBank databases under the accession number: KY652916.1.

### Bioinformatics analysis of CrtE protein

To further explore the molecular features of the *crtE* gene expression product, MEGA7 software was utilized to compare the CrtE protein with the GGPPS of other species. The comparison of these amino acid sequences was shown in Figure 2B, with five highly conserved domains (from I to V), a result similar to that previously reported (Chen et al., 1994). Two of these aspartic acid-rich motifs are located in domains II and V, respectively, where near the N-terminal of the amino acid sequence is known as FARM (first aspartic acid-rich motif) and near the C-terminal is known as SARM (second aspartic acid-rich motif) (Zhang et al., 2021). In the previous crystal structures of prenyltransferase showed that the allylic substrate is bound in the middle of FARM and SARM via magnesium ions (Aaron and Christianson, 2010). In addition, the region which contains FARM and its the upstream five amino acids was defined as CLD (chain length determination region). This region can broadly distinguish the types of prenyltransferase. The CLD region of CrtE is identical to that of *Rhodotorula toruloides* GGPPS and differs from the human GGPPS CLD region by only the first amino acid upstream of FARM. The FARM region of type I GGPPS is DDXXD and its upstream fifth position is an aromatic amino acid with a large side chain and its upstream fourth position is a non-aromatic amino acid; the FARM region of type II GGPPS is DDXXXXD and its upstream fifth and fourth positions are non-aromatic amino acids; the FARM region of type III GGPPS is DDXXXXD and its upstream fifth and fourth positions are non-aromatic amino acids. Therefore CrtE is consistent with the CLD characteristics of type III GGPPS.

**FIGURE 3:**
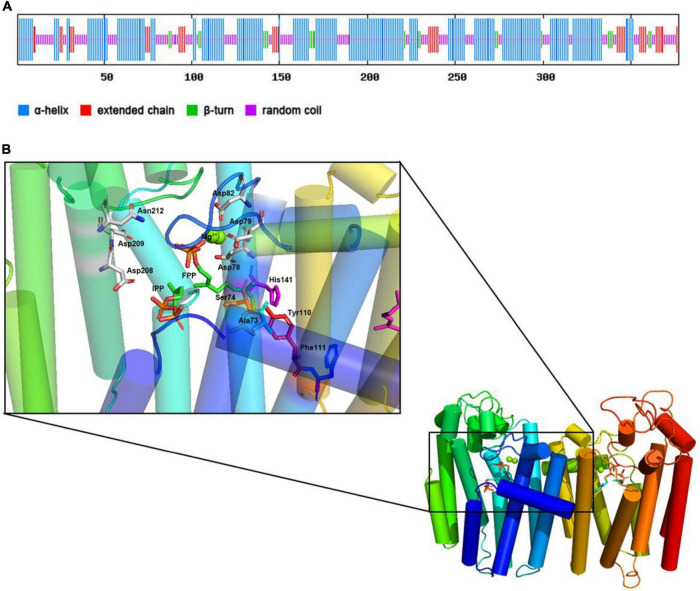
Predicting secondary structure and 3D-model of CrtE. **(A)** Prediction of secondary structure of CrtE. **(B)** 3D-model of the CrtE. The 3D-model prediction of CrtE was performed with the online software SWISS-MODEL. The resulting CrtE 3D-model was visualized in Pymol (The PyMOL Molecular Graphics System, Version 2.0 Schrödinger, LLC.), compared with the *Saccharomyces cerevisiae* GGPPS (PDB ID code 2E8T) model, and ligands FPP (PDB ID code 2E90) and IPP (PDB ID code 2E8U) were added.

Used the SPOPMA online tool for the CrtE protein secondary structure prediction, as shown in Figure 3A, 55.97% of the α-helices, 10.34% of the extended chains, 5.84% of the β-turns, and 27.85% of the random coils. The main secondary structure of the CrtE protein is the α-helix, with the adjacent α-helices linked by other states.

Then, we used the online software SWISS-MODEL to predict the 3D-model of CrtE protein, and the predicted CrtE model has 46.9% sequence identity with the template protein (PDB ID code 6C56) with high reliability. To further explore the enzymatic activity characteristics of CrtE, we compared the CrtE model with the *Saccharomyces cerevisiae* GGPPS model (PDB ID code 2E8T) in the protein visualization software Pymol and embedded the ligands FPP (PDB ID code 2E90), IPP (PDB ID code 2E8U), and Mg^2+^ in CrtE. As shown in Figure 3B, CrtE is a dimeric structure where each subunit is composed of multiple α-helices as well as other secondary structures that link the α-helices in tandem. Eight of these α-helices are spatially arranged vertically, crossed below, and capped above by three short α-helices to form an activity cavity in the core. In addition, a terminal α-helix straddles between the two subunits of dimer and serves to maintain dimerization. We enlarged the activity cavity and the pyrophosphate group of the substrate FPP head was immobilized on the space between FARM and SARM by two Mg^2+^, while IPP was immobilized on the other position.

### Analysis of *in vitro* enzymatic activity of CrtE

**FIGURE 4:**
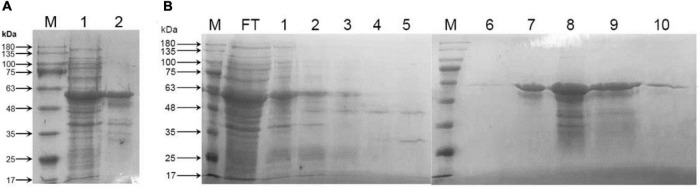
Protein SDS-PAGE analysis. **(A)** SDS-PAGE analysis of total protein in *E. coli* carrying recombinant plasmid pET32a-*crtE*. M: protein marker; 1: soluble proteins in the supernatant; 2: inclusion body proteins in precipitation; **(B)** purification of CrtE from total soluble protein by HisTrap HP column. M: protein marker; FT: flow through; 1–5: wash 1–5; 6–10: elution 1–5. The number on the left-hand side is ColorMixed Protein Marker (Solarbio, Beijing, China). The total protein and CrtE were visualized by Coomassie Brilliant Blue R250 stain.

In order to express CrtE, plasmid pET32a and pET32a-*crtE* was transformed into *E. coli* BL21 (DE3) and expression was induced with IPTG. Following collection of cells for ultrasonic fragmentation, soluble proteins and insoluble precipitates were separated by centrifugation and subjected to SDS-PAGE assay. The total protein and CrtE were visualized by Coomassie Brilliant Blue R250 stain. It could be appeared through (Figure 4A) that the recombinant protein was expressed in the supernatant and inclusion bodies, and more in the supernatant, with a band of about 60 kDa. The molecular weight of the recombinant protein including one Trx⋅tag, one His⋅tag, one S⋅tag, and CrtE was predicted to be 59.06 kDa in ProtParam Tool, consistent with the results obtained by SDS-PAGE.

Recombinant protein pET32a-CrtE carries a His⋅tag and was purified using HisTrap HP column. As shown in Figure 4B the ninth lane corresponding to elution 4 was used to analyze the CrtE enzymatic activity. The recombinant pET32a-CrtE was used to study GGPPS enzymatic properties without cleavage of the His⋅tag, because of the latter has a negligible effect on enzymatic activity (Barbar et al., 2013).

**FIGURE 5:**
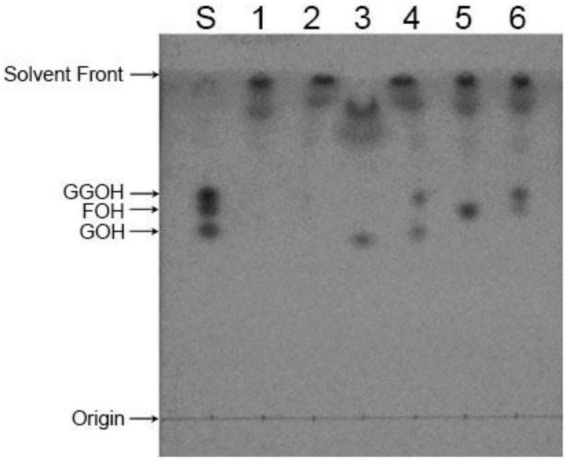
TLC analysis of *in vitro* enzymatic activity reaction products of CrtE. S: GOH, FOH, and GGOH standard mix; 1, 3, and 5: enzymatic reaction system without CrtE protein using DMAPP, GPP, and FPP as substrates, respectively; 2, 4, and 6: enzymatic reaction system containing CrtE protein with DMAPP, GPP, and FPP as substrates, respectively.

The enzymatic activation reaction of CrtE protein occurred in HEPES buffer. The synthesis of the products was observed using three different allylic substrates and IPP as substrates for the reaction. As shown in Figure 5, both three allylic substrates can be used as substrates to synthesize the unique product GGPP. Among them, DMAPP produced the least GGPP as a substrate, which showing a very small spot, utilization of GPP was the second most abundant, and largest number of GGPP was produced using FPP. Furthermore, we can see that a portion of the substrate was remained in the system at the end of the reaction. Because we wanted to see if there was product generation other than GGPP by adding an excess of substrate to the reaction system, the results indicated that a specific product, GGPP, was produced regardless of which allylic substrate was utilized.

### Heterogeneous complementary identification

**FIGURE 6:**
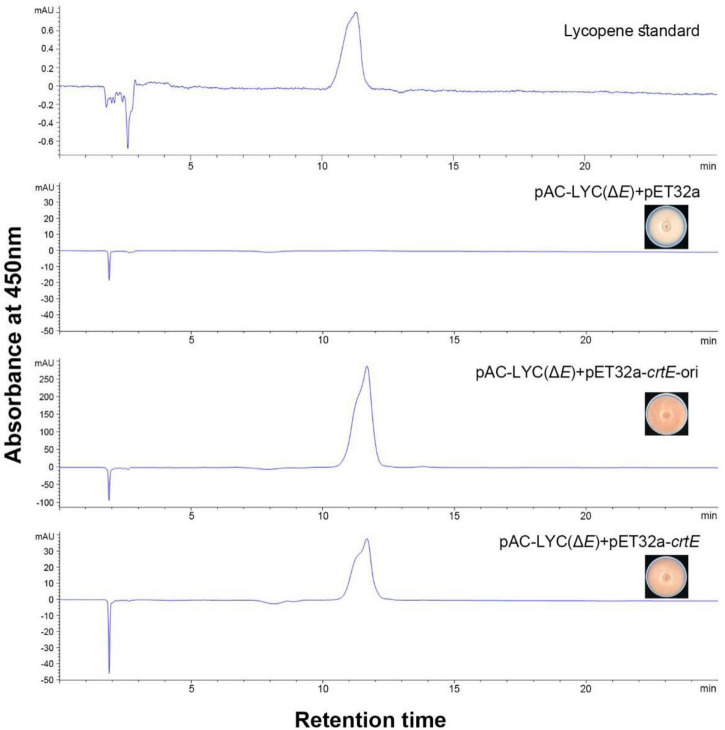
HPLC analysis of carotenoid production of *E. coli* carrying different plasmids. Pigment detection was performed by monitoring absorption at 450 nm.

We successfully constructed plasmid pAC-LYC (Δ*E*) for the identification of the putative GGPPS gene of *S. pararoseus* (Figure 1). In order to test the functional activity of plasmid pAC-LYC (Δ*E*), we cloned the *crtE* gene (GenBank accession number M87280) from *Erwinia herbicola* and ligated it to the expression vector pET32a, which was named pET32a-*crtE*-ori. The colonies of *E. coli* BL21 (DE3) carrying plasmid pAC-LYC (Δ*E*) showed white. When transformed with plasmid pET32a-*crtE*-ori, the colonies changed to red, while the colonies remained white after transforming the empty vector pET32a (as shown in Figure 6). And HPLC analysis showed that plasmid pAC-LYC (Δ*E*) and pET32a-*crtE*-ori co-expressing strains accumulated lycopene (as shown in Figure 6). To sum up, these results suggest that plasmid pAC-LYC (Δ*E*) can exert its functional activity in *E. coli* BL21 (DE3).

The above complementary experiments are used to identified the functional of the *crtE* gene of *S. pararoseus*. The color of strains changed from white to red after transformation of plasmid pET32a-*crtE* into *E. coli* BL21 (DE3) carrying plasmid pAC-LYC (Δ*E*). HPLC analysis showed that the accumulating lycopene of the recombinant strain carrying pAC-LYC (Δ*E*) transformed with pET32a-*crtE* plasmid (as shown in Figure 6). The above results indicate that the *crtE* gene encodes a GGPPS functional protein in *E. coli* and its metabolites synthesize lycopene under the action of phytoene synthase (CrtB) and phytoene desaturase (CrtI). Furthermore, we found that *E. coli* harboring the plasmid pAC-LYC (Δ*E*) and *S. pararoseus crtE* produces fewer pigments than that of harboring pAC-LYC (Δ*E*) and *E. herbicola crtE*. A similar phenomenon has been observed in the process of functional characterization of the gene encoding GGPPS from *Dunaliella bardawil* (Liang et al., 2015). The expression of *DbGGPS* in *E. coli* led to less accumulation of the lycopene than that of the gene *crtE* from *Erwinia uredovora*. This could be due to the fact that CrtE of *E. herbicola* has a higher expression level than CrtE of *S. pararoseus* in *E. coli* or has a stronger enzymatic activity. In addition, certain characteristics of the protein itself or the culture conditions of *E. coli* may be the decisive factors of enzymatic activity.

## Discussion

*Sporobolomyces pararoseus*, *Rhodosporidium toruloides*, *Rhodotorula glutinis*, and *Xanthophyllomyces dendrorhous*, among others, are red yeasts that are of great interest because of their high production of carotenoids and lipids, short life cycle, and ease of cultivation (Chaiyaso and Manowattana, 2018). In previous studies, the phytoene desaturase gene *crtI* (Guo et al., 2015) and the bifunctional lycopene cyclase/phytoene synthase gene *crtYB* (Guo et al., 2014) in the carotenogenic pathway of red yeast have been successfully cloned and characterized, whereas studies encoding the GGPPS gene *crtE* are rarely reported. In this study, we cloned the *crtE* gene of *S. pararoseus* NGR isolated from strawberry fruits based on the results of transcriptome sequencing, and investigated the mechanism of enzymatic activity of its expression product in depth.

Based on the characteristics of CLD, CrtE was classified as type III GGPPS. The fourth and fifth positions upstream of the FARM of CrtE are small residue amino acids (Ser74 and Ala73) that could not form a spatial block at these positions for product chain length extension as in the case of type I GGPPS and FPPS (Wang and Ohnuma, 1999). In a study of the mechanism of product chain length extension of yeast GGPPS, it was shown that Tyr107, Phe108, and His139 are key amino acids that prevent the continued extension of type III GGPPS, and mutation of these sites with Ala resulted in product chain length extension up to C70 (Chang et al., 2006). These three positions correspond to Tyr110, Phe111, and His141 in CrtE. In Figure 3B, it could be seen that Tyr110 and His141 are spatially parallel to the fifth amino acid upstream of the FARM region, and Phe111 is below them, therefore these positions may be the key sites that limit the continued extension of GGPP in CrtE. In addition, the yeast Ser71 (the fourth amino acid upstream of FARM) was mutated to Tyr and the product was completely changed to FPP (Chang et al., 2006). Similar to the CLD feature of TgFPPS, its fourth position upstream of FARM is Phe and fifth position is Cys, thus exhibiting FPPS/GGPPS bifunctional enzymatic activity (Li et al., 2012). Mutation of the upstream fourth position of type III GGPPS to a medium chain length amino acid or mutation of the upstream fifth amino acid to an aromatic amino acid could convert it to FPP/GGPP bifunctional activity, a conjecture to be further tested.

The results of *in vitro* enzymatic activity experiments showed that CrtE synthesized the sole product GGPP regardless of the allylic substrate utilized. This is consistent with the above conjecture that it is possible that Tyr110, Phe111, and His141 by CrtE act as spatial blocks to keep GGPP from continuing to extend. In addition, more GGPP was produced using FPP in the three allylic substrates, this phenomenon is consistent with the results in GGPPSs of human (Kavanagh et al., 2006) and *Choristoneura fumiferana* (Barbar et al., 2013) and is in line with typical type III GGPPS. Therefore, there are enzymes in *S. pararoseus* that are specifically responsible for the production of FPP, providing substrates for metabolites such as GGPP and isoprenylated proteins.

*E. coli* cannot synthesize GGPP by itself, but can synthesize and accumulate FPP (Umeno et al., 2005), so heterologous complementation experiments can be performed in *E. coli* to verify the function of CrtE. The carotenoid synthesis genes *crtE*, *crtI*, and *crtB* from *E. herbicola* were expressed in *E. coli* in the vector pAC-LYC to synthesize lycopene, which gives the colony its red color (Cunningham et al., 1994). The system was used in combination with HPLC for carotenoid detection and observation of colony to perform functional characterization of the heterologous phytoene desaturase gene, the bifunctional lycopene cyclase/phytoene synthase gene and the GGPPS gene. When the *crtE* gene of *S. pararoseus* NGR was co-expressed with pAC-LYC (Δ*E*) in *E. coli*, the colony became red due to the synthesis and accumulation of lycopene, indicating that the *crtE* gene encodes the synthesis of GGPPS functional protein *in vivo*. The low expression of the *S. pararoseus crtE* gene in *E. coli* may be improved by optimizing the codon. The yield of metabolites is generally enhanced by overexpression of homologous genes in some engineered strains, thus allowing the elimination of some sequence optimization steps, such as the promotion of biomass accumulation and carotenoid production after overexpression of carotenoid synthesis-related genes in *X. dendrorhous* and *Corynebacterium glutamicum* (Heider et al., 2014; Chi et al., 2015).

In summary, we have identified the *crtE* gene encoding functional GGPPS and investigated the enzymatic activity mechanism of CrtE protein in depth, which provides a molecular theoretical basis for the subsequent construction of carotenoid-producing *S. pararoseus* using genetic engineering biotechnology, and is conducive to providing better strains for industry with high carotenoid production.

## Conclusion

The putative gene *crtE* encoding GGPPS in *S. pararoseus* was functionally characterized by heterologous complementation experiments, and the gene expression product CrtE was purified and characterized for enzymatic activity. CrtE was classified as type III GGPPS and catalyzed the condensation reaction of FPP with IPP to produce the sole product GGPP. The predicted three-dimensional structure of CrtE was analyzed to correlate its amino acid sequence with the enzymatic activity characteristics. This will facilitate the understanding of GGPPS in *S. pararoseus* and provide a theoretical basis for the construction of engineered strains used in industrial production.

## Data availability statement

The datasets presented in this study can be found in online repositories. The names of the repository/repositories and accession number(s) can be found in the article/supplementary material.

## Author contributions

JY performed the experiments, analyzed the data, and wrote the draft manuscript. CJL designed the experiments and participated in the manuscript writing and revision. NZ provided the experimental materials and multiple technical supports. CWL and YW provided assistant in all the experiment preparations. BL conceived, designed, supervised the experiment, and acquired funding. All authors approved the final version of the manuscript.

## Footnotes

1
https://web.expasy.org/protparam/

2
https://swissmodel.expasy.org/

## References

[B1] Aaron J. A. Christianson D. W. (2010). Trinuclear metal clusters in catalysis by terpenoid synthases. *Pure Appl. Chem.* 82 1585–1597. 10.1351/pac-con-09-09-37 21562622 PMC3090183

[B2] Ames B. N. Shigenaga M. K. Hagen T. M. (1993). Oxidants, antioxidants, and the degenerative diseases of aging. *Proc. Natl. Acad. Sci. U. S. A.* 90 7915–7922. 10.1073/pnas.90.17.7915 8367443 PMC47258

[B3] Barbar A. Couture M. Sen S. E. Béliveau C. Nisole A. Bipfubusa M. (2013). Cloning, expression and characterization of an insect geranylgeranyl diphosphate synthase from *Choristoneura fumiferana*. *Insect Biochem. Mol. Biol.* 43 947–958. 10.1016/j.ibmb.2013.07.004 23907071

[B4] Breitenbach J. Visser H. Verdoes J. C. van Ooyen A. J. Sandmann G. (2011). Engineering of geranylgeranyl pyrophosphate synthase levels and physiological conditions for enhanced carotenoid and astaxanthin synthesis in *Xanthophyllomyces dendrorhous*. *Biotechnol. Lett.* 33 755–761. 10.1007/s10529-010-0495-2 21165672

[B5] Cakir E. Cakir U. Tayman C. Turkmenoglu T. T. Gonel A. Turan I. O. (2020). Favorable effects of astaxanthin on brain damage due to ischemia- reperfusion injury. *Comb. Chem. High Throughput Screen.* 23 214–224. 10.2174/1386207323666200219121600 32072893

[B6] Chaiyaso T. Manowattana A. (2018). Enhancement of carotenoids and lipids production by oleaginous red yeast *Sporidiobolus pararoseus* KM281507. *Prep. Biochem. Biotechnol.* 48 13–23. 10.1080/10826068.2017.1381620 29035150

[B7] Chang T. H. Guo R. T. Ko T. P. Wang A. H. Liang P. H. (2006). Crystal structure of type-III geranylgeranyl pyrophosphate synthase from *Saccharomyces cerevisiae* and the mechanism of product chain length determination. *J. Biol. Chem.* 281 14991–15000. 10.1074/jbc.M512886200 16554305

[B8] Chen A. Kroon P. A. Poulter C. D. (1994). Isoprenyl diphosphate synthases: Protein sequence comparisons, a phylogenetic tree, and predictions of secondary structure. *Protein Sci.* 3 600–607. 10.1002/pro.5560030408 8003978 PMC2142870

[B9] Chi S. He Y. Ren J. Su Q. Liu X. Chen Z. (2015). Overexpression of a bifunctional enzyme, CrtS, enhances astaxanthin synthesis through two pathways in *Phaffia rhodozyma*. *Microb. Cell Fact.* 14:90. 10.1186/s12934-015-0279-4 26081576 PMC4470029

[B10] Costa-Rodrigues J. Pinho O. Monteiro P. R. R. (2018). Can lycopene be considered an effective protection against cardiovascular disease?. *Food Chem.* 245 1148–1153. 10.1016/j.foodchem.2017.11.055 29287334

[B11] Cunningham F. X. Jr. Sun Z. Chamovitz D. Hirschberg J. Gantt E. (1994). Molecular structure and enzymatic function of lycopene cyclase from the cyanobacterium *Synechococcus* sp strain PCC7942. *Plant Cell* 6 1107–1121. 10.1105/tpc.6.8.1107 7919981 PMC160505

[B12] Guo W. Liu Y. Yan X. Liu M. Tang H. Liu Z. (2015). Cloning and characterization of a phytoene dehydrogenase gene from marine yeast *Rhodosporidium diobovatum*. *Antonie Van Leeuwenhoek* 107 1017–1027. 10.1007/s10482-015-0394-6 25627014

[B13] Guo W. Tang H. Zhang L. (2014). Lycopene cyclase and phytoene synthase activities in the marine yeast *Rhodosporidium diobovatum* are encoded by a single gene *crtYB*. *J. Basic Microbiol.* 54 1053–1061. 10.1002/jobm.201300920 24677129

[B14] Hara K. Y. Morita T. Mochizuki M. Yamamoto K. Ogino C. Araki M. (2014). Development of a multi-gene expression system in *Xanthophyllomyces dendrorhous*. *Microb. Cell Fact.* 13:175. 10.1186/s12934-014-0175-3 25471659 PMC4264253

[B15] Heider S. A. Wolf N. Hofemeier A. Peters-Wendisch P. Wendisch V. F. (2014). Optimization of the IPP precursor supply for the production of lycopene, decaprenoxanthin and astaxanthin by *Corynebacterium glutamicum*. *Front. Bioeng. Biotechnol.* 2:28. 10.3389/fbioe.2014.00028 25191655 PMC4138558

[B16] Kavanagh K. L. Dunford J. E. Bunkoczi G. Russell R. G. G. Oppermann U. (2006). The crystal structure of human geranylgeranyl pyrophosphate synthase reveals a novel hexameric arrangement and inhibitory product binding. *J. Biol. Chem.* 281 22004–22012. 10.1074/jbc.M602603200 16698791

[B17] Kot A. M. Błażejak S. Gientka I. Kieliszek M. Bryś J. (2018). Torulene and torularhodin: “new” fungal carotenoids for industry?. *Microb. Cell Fact.* 17:49. 10.1186/s12934-018-0893-z 29587755 PMC5870927

[B18] Kot A. M. Błażejak S. Kurcz A. Gientka I. Kieliszek M. (2016). *Rhodotorula glutinis*-potential source of lipids, carotenoids, and enzymes for use in industries. *Appl. Microbiol. Biotechnol.* 100 6103–6117. 10.1007/s00253-016-7611-8 27209039 PMC4916194

[B19] Li C. Zhang N. Li B. Xu Q. Song J. Wei N. (2017). Increased torulene accumulation in red yeast *Sporidiobolus pararoseus* NGR as stress response to high salt conditions. *Food Chem.* 237 1041–1047. 10.1016/j.foodchem.2017.06.033 28763948

[B20] Li C. Zhang N. Song J. Wei N. Li B. Zou H. (2016). A single desaturase gene from red yeast *Sporidiobolus pararoseus* is responsible for both four- and five-step dehydrogenation of phytoene. *Gene* 590 169–176. 10.1016/j.gene.2016.06.042 27346167

[B21] Li C. J. Zhao D. Li B. X. Zhang N. Yan J. Y. Zou H. T. (2020). Whole genome sequencing and comparative genomic analysis of oleaginous red yeast *Sporobolomyces pararoseus* NGR identifies candidate genes for biotechnological potential and ballistospores-shooting. *BMC Genomics* 21:181. 10.1186/s12864-020-6593-1 32093624 PMC7041287

[B22] Li Z. H. Cintrón R. Koon N. A. Moreno S. N. (2012). The N-terminus and the chain-length determination domain play a role in the length of the isoprenoid product of the bifunctional *Toxoplasma gondii* farnesyl diphosphate synthase. *Biochemistry* 51 7533–7540. 10.1021/bi3005335 22931372 PMC4618988

[B23] Liang M. H. Liang Y. J. Jin H. H. Jiang J. G. (2015). Characterization and functional identification of a gene encoding geranylgeranyl diphosphate synthase from *Dunaliella bardawil*. *J. Agric. Food Chem.* 63 7805–7812. 10.1021/acs.jafc.5b02732 26289929

[B24] Moise A. R. Al-Babili S. Wurtzel E. T. (2014). Mechanistic aspects of carotenoid biosynthesis. *Chem. Rev.* 114 164–193. 10.1021/cr400106y 24175570 PMC3898671

[B25] Mussagy C. U. Winterburn J. Santos-Ebinuma V. C. Pereira J. F. B. (2019). Production and extraction of carotenoids produced by microorganisms. *Appl. Microbiol. Biotechnol.* 103 1095–1114. 10.1007/s00253-018-9557-5 30560452

[B26] Mustra Rakic J. Wang X. D. (2020). Role of lycopene in smoke-promoted chronic obstructive pulmonary disease and lung carcinogenesis. *Arch. Biochem. Biophys.* 689:108439. 10.1016/j.abb.2020.108439 32504553

[B27] Nagel R. Schmidt A. Peters R. J. (2019). Isoprenyl diphosphate synthases: The chain length determining step in terpene biosynthesis. *Planta* 249 9–20. 10.1007/s00425-018-3052-1 30467632

[B28] Ojulari O. V. Lee S. G. Nam J. O. (2020). Therapeutic effect of seaweed derived xanthophyl carotenoid on obesity management; overview of the last decade. *Int. J. Mol. Sci.* 21:2502. 10.3390/ijms21072502 32260306 PMC7177665

[B29] Schmidt A. Wächtler B. Temp U. Krekling T. Séguin A. Gershenzon J. (2010). A bifunctional geranyl and geranylgeranyl diphosphate synthase is involved in terpene oleoresin formation in *Picea abies*. *Plant Physiol.* 152 639–655. 10.1104/pp.109.144691 19939949 PMC2815902

[B30] Shen H. J. Cheng B. Y. Zhang Y. M. Tang L. Li Z. Bu Y. F. (2016). Dynamic control of the mevalonate pathway expression for improved zeaxanthin production in *Escherichia coli* and comparative proteome analysis. *Metab. Eng.* 38 180–190. 10.1016/j.ymben.2016.07.012 27474352

[B31] Umeno D. Tobias A. V. Arnold F. H. (2005). Diversifying carotenoid biosynthetic pathways by directed evolution. *Microbiol. Mol. Biol. Rev.* 69 51–78. 10.1128/mmbr.69.1.51-78.2005 15755953 PMC1082795

[B32] Vandermoten S. Haubruge E. Cusson M. (2009). New insights into short-chain prenyltransferases: Structural features, evolutionary history and potential for selective inhibition. *Cell Mol. Life Sci.* 66 3685–3695. 10.1007/s00018-009-0100-9 19633972 PMC11115643

[B33] Wang K. Ohnuma S. (1999). Chain-length determination mechanism of isoprenyl diphosphate synthases and implications for molecular evolution. *Trends Biochem. Sci.* 24 445–451. 10.1016/s0968-0004(99)01464-4 10542413

[B34] Wei C. Wu T. Ao H. Qian X. Wang Z. Sun J. (2020). Increased torulene production by the red yeast, *Sporidiobolus pararoseus*, using citrus juice. *Prep. Biochem. Biotechnol.* 50 66–73. 10.1080/10826068.2019.1663533 31502910

[B35] Yang Y. Yatsunami R. Ando A. Miyoko N. Fukui T. Takaichi S. (2015). Complete biosynthetic pathway of the C50 carotenoid bacterioruberin from lycopene in the extremely halophilic archaeon *Haloarcula japonica*. *J. Bacteriol.* 197 1614–1623. 10.1128/jb.02523-14 25712483 PMC4403650

[B36] Zhang C. Liu H. Zong Y. Tu Z. Li H. (2021). Isolation, expression, and functional analysis of the geranylgeranyl pyrophosphate synthase (GGPPS) gene from *Liriodendron tulipifera*. *Plant Physiol. Biochem.* 166 700–711. 10.1016/j.plaphy.2021.06.052 34214780

